# Surgical Lymph Node Biopsy for the Diagnosis of Lymphoma: A Case Report

**DOI:** 10.7759/cureus.49063

**Published:** 2023-11-19

**Authors:** Chih Ching Wu, Ethan Leng, Trevor F Killeen, Erik B Faber, James V Harmon

**Affiliations:** 1 Department of Surgery, University of Minnesota Medical School, Minneapolis, USA; 2 Department of Laboratory Medicine and Pathology, University of Minnesota Medical School, Minneapolis, USA

**Keywords:** diffuse large b-cell lymphoma (dlbcl), follicular lymphoma, fine-needle aspiration, lymph node excision, core needle biopsy, lymphoma

## Abstract

We report the diagnosis, treatment, and outcomes of a 52-year-old woman who originally presented to her primary care provider with adenopathy. Core needle biopsy (CNB) was inconclusive as it could not distinguish between follicular and diffuse large B-cell lymphomas (DLBCLs). A left axillary surgical lymph node biopsy was performed and demonstrated that the patient had a DLBCL arising from grade 3 follicular lymphoma. We discuss the limitations of CNB and the value of surgical lymph node biopsy in the diagnosis of lymphoma. The patient recovered from the biopsy without complications, and chemotherapy was initiated after the procedure. The patient has now remained in complete remission at 22 months.

## Introduction

Syrykh et al. suggested that core needle biopsy (CNB) provides a definitive diagnosis of suspected lymphoma in 92% of patients, while surgical excision makes possible a definitive diagnosis in 98% of patients; unless strictly contraindicated, surgical lymph node biopsy, rather than CNB or fine needle aspiration (FNA), is considered essential for diagnosis [1]. Among the most common types of non-Hodgkin lymphoma (NHL) are diffuse large B-cell lymphoma (DLBCL), which accounts for up to 32% of all NHL cases, and follicular lymphoma, which constitutes up to 17% [2]. NHL often arises in the lymph nodes, but it may present as extranodal masses [3].

Open lymph node biopsy is a well-established technique for the diagnosis of lymphoma. Alternatives to open lymph node biopsy include CNB and FNA. However, CNB and FNA may result in insufficient tissue samples for diagnosis. Moreover, CNB and FNA have been associated with up to 18% misidentification of NHL subtypes [1,4]. We discuss a patient’s history, radiologic findings, surgical technique, potential complications, and ultimate diagnosis and highlight the limitations of CNB and FNA for the evaluation of lymphoma. In this case, the preliminary assessment of CNB was follicular lymphoma; however, a careful consideration of the biopsy specimen led to the realization that the CNB was insufficient for definitive diagnosis, and an excisional biopsy was requested.

## Case presentation

**Figure 1:**
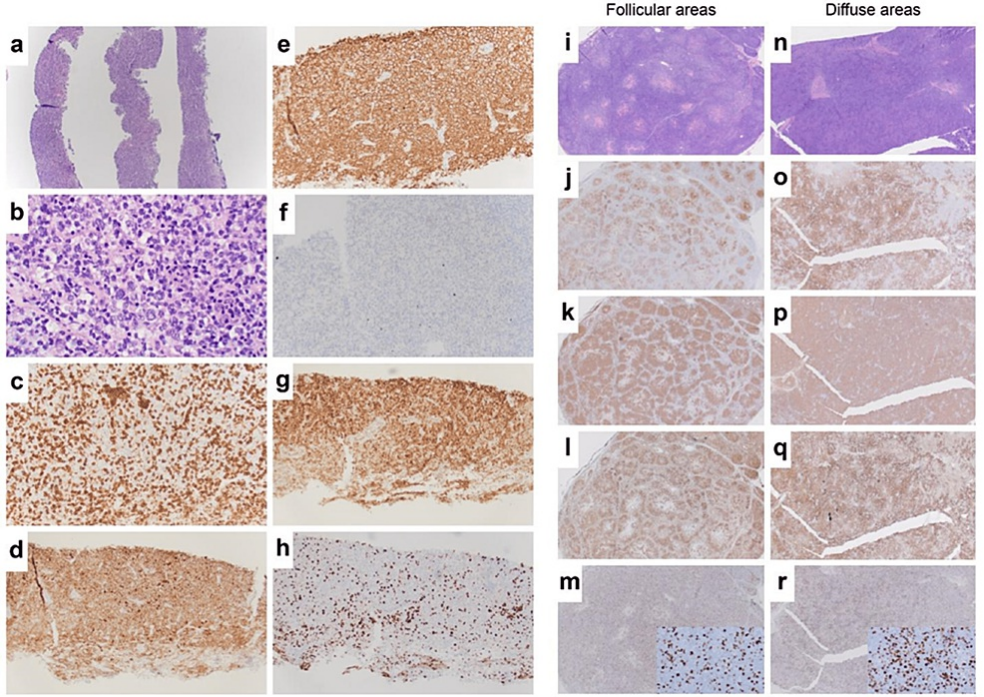
Surgical pathology Core needle biopsy (a–h) and excisional axillary lymph node biopsy (i–r). i–m of the excisional lymph node biopsy demonstrates a histologic pattern consistent with follicular lymphoma, while n–r of the excisional lymph node biopsy demonstrates a histologic pattern consistent with the diffuse large B-cell lymphoma (DLBCL). (a) 4x and (b) 50x H&E; (c) 20x CD3 stains reactive infiltrating T cells; (d) 20x CD10 stains B cells of follicle center origin (including lymphoma cells in follicular lymphoma and DLBCL); (e) 20x cd20+, marker for B lymphocytes; (f) 20x CD21+, marker for follicular dendritic cells; (g) 20x BCL-2, an anti-apoptotic protein detected in follicular lymphomas and associated with BCL-2 overexpression due to t(14;18) translocations; (h) 20x Ki-67, marker of cell proliferation; (i) 2x H&E follicular areas; (j) 2x CD10+; (k) 2x CD20+; (l) 2x BCL-2; (m) 2x (insert 40x) Ki-67; (n) 2x H&E diffuse large B cell; (o) 2x CD10+; (p) 2x CD20+; (q) 2x BCL-2; (r) 2x (insert 40x) Ki-67.

A 52-year-old woman presented to her primary care provider with a three-month history of left-sided axillary lymphadenopathy, without fevers, weight loss, or night sweats. The patient also reported more recent left supraclavicular lymphadenopathy in addition to the axillary lymphadenopathy. She was on methotrexate and sulfasalazine for psoriatic arthritis. On examination, the left axilla demonstrated multiple palpable enlarged lymph nodes. The white blood cell count was 8.4x10^9^/L, platelet count was 315x10^9^/L, alanine aminotransferase was 47 U/L, aspartate aminotransferase was 35 U/L, and calcium was 9.2 mg/dl. Diagnostic imaging studies included a bilateral mammogram with left breast and axillary ultrasound, left neck soft tissue ultrasound, chest X-ray, and chest and abdominal computed tomography (CT). CT demonstrated extensive left axillary lymphadenopathy, and the largest lymph node was 6.5 cm × 3.9 cm × 3.4 cm. The patient underwent percutaneous core needle biopsy (CNB) (four needle tissue cores ranging from 0.2 to 0.8 cm in length and 0.1 cm in diameter), which was interpreted to favor follicular lymphoma (Figure 1a-1h). For definitive diagnosis and grading, an excisional lymph node biopsy was recommended by the pathologist.

**Figure 2:**
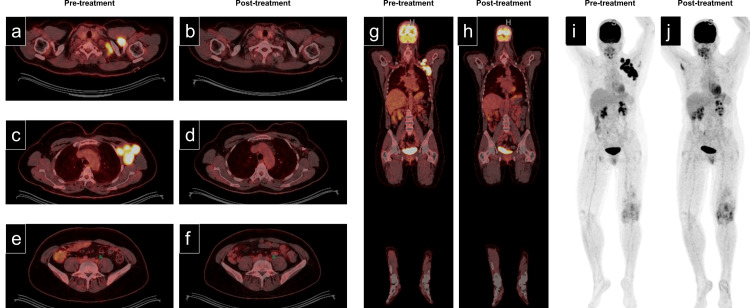
Radiographic positron emission tomography/computed tomography (PET/CT) imaging Positron emission tomography/computed tomography (PET/CT) images. Corresponding pairs of images (a, c, e: pretreatment; b, d, f: posttreatment) were acquired before and after treatment. (a, b) supraclavicular, (c, d) axillary, (e, f) lower abdomen/pelvis. The green arrow in images e and f demonstrates decreased size and fluorodeoxyglucose (FDG) avidity after treatment. No new areas of focal avidity were noted after treatment. (g, h) Pre- and posttreatment coronal views. (i, j) Pre- and posttreatment maximum intensity projection (MIP) images. The focus of the FDG uptake involving the left knee was attributed to pre-existing inflammatory arthritis.

The clinical differential diagnosis included follicular lymphoma, DLBCL, or germinal center B-cell lymphoma subtypes. The patient then underwent axillary lymph node scanning by [18F] fluorodeoxyglucose (FDG)-positron emission tomography (PET)/computed tomography (CT) [3,5]. The PET scan confirmed significant FDG avidity of the enlarged left-sided nodes identified on the preceding CT (Figure 2a, 2c, 2e, 2g). The maximum standard uptake value (SUV max) was >10 for the axillary nodes. Numerous mesenteric and retroperitoneal lymph nodes demonstrated borderline enlargement with increased avidity, with the largest lymph node having in-plane dimensions of 0.9 × 0.6 cm and an SUV max of 3.1 (Figure 2c).

**Figure 3:**
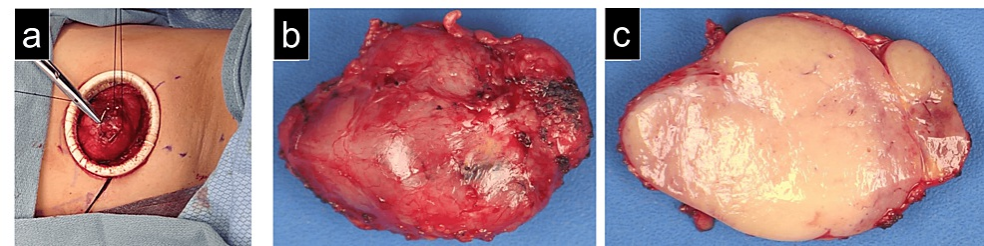
Intraoperative and gross pathologic images (a) Surgical biopsy of the enlarged left axillary lymph node. Placement of a 0 silk suture assists in elevating the lymph node during dissection. An Alexis wound protector (Applied Medical, Rancho Santa Margarita, CA) facilitates exposure and gentle tissue handling. All afferent and efferent lymphatic channels were controlled using surgical clips to avoid postoperative lymphocele formation. (b) Gross surface of the enlarged, firm, tan-red lymph node, measuring 5.2 cm x 3.6 cm x 2.5 cm. (c) The cut surface of the lymph node demonstrated a nodular expansion of uniform tan tissue without hemorrhage or necrosis.

This was thought to represent the involvement of infradiaphragmatic nodes, indicating a stage III disease [6]. A bone marrow biopsy demonstrated a normal distribution of trilineage hematopoiesis and no morphologic or immunophenotypic evidence of B-cell lymphoma. As the final histologic distinction could not be made on CNB, surgery was consulted, and a diagnostic left axillary lymph node biopsy was performed (Figure 3). The patient's left arm was supported with an arm sling to avoid nerve injury. A curvilinear incision was made along the inferior aspect of the hair-bearing axilla to facilitate the exposure. The largest lymph node was suspended by a figure of eight stitch using 0 silk suture to aid in the identification of the afferent and efferent lymphatics. An Alexis O wound protector (Applied Medical, Rancho Santa Margarita, CA) was placed to protect the skin edges from surgical trauma. Surgical clips (Braun, Tuttlingen, Germany) controlled the afferent and efferent lymphatic channels to avoid a lymphocele. The excised lymph node was submitted fresh to the pathology laboratory for lymphoma work-up. The deep soft tissue and skin were closed in layers, and Exofin skin sealant (Chemence Medical, Alpharetta, GA) was used to reapproximate the epidermis and minimize wound infection.

**Figure 4:**
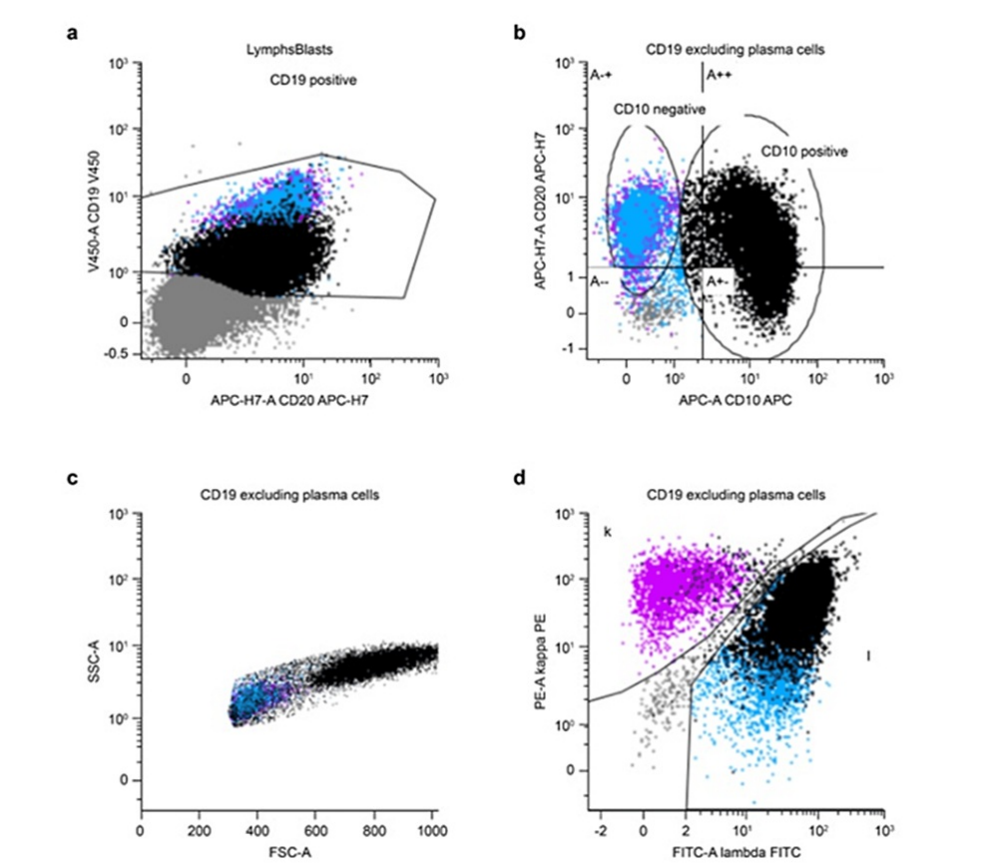
Flow cytometry Flow cytometry scattergram plots of the B-cell population. (a) Lymphoma cells (denoted as black on the scatter plot) demonstrated lower levels of CD19 positivity than normal B cells (denoted as blue and magenta on the scatter plot). (b) Lymphoma and normal cells were defined by expression on CD19 and/or CD20 positivity, and then gated into the CD10+ and CD10- populations, respectively. (c) Lymphoma cells demonstrated overall higher levels of forward scatter and slightly higher levels of side scatter, suggesting a larger size and cytoplasmic complexity, respectively, compared to normal cells. (d) Lymphoma cells demonstrated nearly exclusively lambda light chain restriction, while normal B cells demonstrated a mixed proportion of lambda and kappa light chain-expressing cells.

The histological evaluation demonstrated 90% lymph node effacement by DLBCL and high-grade follicular lymphoma (grade 3A) involvement in the remaining 10% (Figure 1 i-r). The germinal centers expressed MYC and BCL2, and concurrent flow cytometry (Figure 4) showed 19% CD10-positive lambda-monotypic B cells. Fluorescence in situ hybridization (FISH) results demonstrated complex signal patterns consistent with the presence of a hyperploid or polyploid complement. BCL2 rearrangement, a common finding in NHL, was also observed. However, as there was no evidence of MYC rearrangement or MYC-IGH fusion, this was not a "double-hit" lymphoma.

Prior to the initiation of chemotherapy, an echocardiogram was performed, demonstrating an ejection fraction of 60-65% and no significant valvular or chamber abnormalities. The International Prognostic Index assigned this patient to a "low-risk group," with a five-year overall survival of 82%. Systemic chemotherapy was initiated with R-CHOP (rituximab, cyclophosphamide, vincristine, doxorubicin, and prednisone). An interim PET scan after two cycles demonstrated a complete response. After six cycles of R-CHOP, the patient remained in remission at 22 months based on PET scans (Figure 2b, 2d, 2f, 2h).

## Discussion

Among the selected patients, CNB provides access to deep enlarged lymph nodes using ultrasound guidance in up to 81% of patients [7]. Enlarged lymph nodes in patients with NHL are almost always pathologic as opposed to reactive; a size >2 cm alone is adequate for the selection of lymph node biopsy. Other factors indicative of malignancy include persistence for >four weeks and increasing dimensions [8]. Ultrasound can be helpful in correctly identifying a malignant lymph node; the presence of a fatty hilum is characteristic of benign lymph nodes on ultrasound examination [9]. Hypoechoic features, internal necrosis, mixed vascularity, and loss of a fatty hilum are the ultrasound characteristics of malignant lymph nodes [10]. In addition, compared to benign or reactive lymph nodes, malignant lymph nodes are generally round, with a long-axis-to-short-axis ratio of <2 [11]. Malignant lymph nodes also have more peripheral than hilar vascularity [12,13]. In the patient, of the many lymph nodes available for biopsy, the largest left axillary lymph node was chosen, partly due to its size and accessibility to the surgical site, as well as its metabolic activity seen on PET/CT. It is important to note that this patient’s lymph node had been previously sampled by CNB without adequate tissue for diagnosis.

Although complications from lymph node excisions are rare, CNB and FNA are sometimes performed to avoid the morbidity associated with surgery. However, various studies have demonstrated that CNB and FNA can be insufficient for characterizing lymphoma. According to the National Comprehensive Cancer Network (NCCN) guidelines for lymphoma, FNA alone is not suitable for initial diagnosis, and CNB is suboptimal but may be indicated in certain clinical circumstances, such as patients with comorbidities who may be unable to tolerate required anesthesia, as well as those who have lymph node involvement in anatomic locations which are not readily amenable to excisional lymph node biopsy, including deep spaces like the retroperitoneum [14]. Surgical lymph node biopsy complications, which are infrequent, include hematoma, seroma, lymphocele, damage to the surrounding structures, and wound infections [15].

There are significant diagnostic benefits of lymph node biopsy over CNB and FNA, which often yield insufficient tissues for lymphoma evaluation. Sampling errors using CNB and FNA may result in misdiagnoses and incomplete subclassification of lymphoma. Prior studies document that CNB provided the correct lymphoma diagnosis in only 66% of patients, provided an incorrect lymphoma subtype in 18% of patients, yielded inconclusive results in 14% of patients, and misidentified the tissue as benign in 2% of patients [16-19].

The patient’s initial CNB demonstrated lymphoma of follicular origin, but the pathologist was concerned that the diagnosis might be incomplete; thus, a surgical lymph node biopsy was performed. Histological evaluation of the surgical lymph node biopsy led to the correct diagnosis and proper treatment of DLBCL arising in a background of follicular lymphoma. Treatment of low-grade follicular lymphoma can be limited to rituximab immunotherapy alone. However, DLBCL requires R-CHOP therapy. The estimated five-year survival rate for low-grade follicular lymphoma is 94%, whereas that for DLBCL is 65% [20]. Selected low-grade lymphomas can simply be observed or treated with single-agent rituximab. However, in addition to rituximab, some first-line treatment options for low-grade follicular lymphoma include bendamustine, obinutuzumab, CHOP, CVP (cyclophosphamide, vincristine, prednisone), or lenalidomide [14]. By contrast, CHOP alone or in combination with radiation therapy is the most common first-line treatment for DLBCL.

## Conclusions

The patient underwent surgical biopsy of a large lymph node from the left axilla and received chemotherapy two weeks later without surgical complications. CNB and FNA can result in an incomplete or inaccurate diagnosis of lymphoma subtypes and potentially lead to suboptimal treatment regimens. This case report illustrates the value of an excisional lymph node biopsy when the evaluation of core needle biopsy samples fails to provide a diagnosis or a specific classification of lymphoma.
